# Novel cancer therapies for advanced cutaneous melanoma: The added value of radiomics in the decision making process–A systematic review

**DOI:** 10.1002/cam4.2709

**Published:** 2020-01-17

**Authors:** Antonino Guerrisi, Emiliano Loi, Sara Ungania, Michelangelo Russillo, Vicente Bruzzaniti, Fulvia Elia, Flora Desiderio, Raffaella Marconi, Francesco Maria Solivetti, Lidia Strigari

**Affiliations:** ^1^ Radiology and Diagnostic Imaging Unit Department of Clinic and Dermatological Research San Gallicano Dermatological Institute IRCCS Rome Italy; ^2^ Medical Physics and Expert Systems Laboratory Department of Research and Advanced Technologies Istituti Fisioterapici Ospitalieri ‐Regina Elena Institute IRCCS Rome Italy; ^3^ Medical Oncology Unit 1 Department of Clinic and Cancer Research Regina Elena Institute IRCCS Rome Italy

**Keywords:** cutaneous melanoma, immunotherapy, precision medicine, radiomics, texture analysis

## Abstract

Advanced malignant melanoma represents a public health matter due to its rising incidence and aggressiveness. Novel therapies such as immunotherapy are showing promising results with improved progression free and overall survival in melanoma patients. However, novel targeted and immunotherapies could generate atypical patterns of response which are nowadays a big challenge since imaging criteria (ie Recist 1.1) have not been proven to be always reliable to assess response. Radiomics and in particular texture analysis (TA) represent new quantitative methodologies which could reduce the impact of these limitations providing most robust data in support of clinical decision process. The aim of this paper was to review the state of the art of radiomics/TA when it is applied to the imaging of metastatic melanoma patients.

## Graphical abstract

Novel cancer therapies are showing promising results with improved progression free and overall survival in patients with advanced melanoma. Radiomics represents new emerging quantitative methodologies that provide most robust data to support clinical decision in particular when atypical treatment response is present in these patients. The aim of this paper was to review the state of the art of radiomics when it is applied to the imaging of malignant melanoma patients.

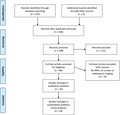

## 1 INTRODUCTION

In the last 50 years, the incidence of malignant melanoma has increased faster than almost any other cancer and it represents a public health matter in many countries due to its high rate of mortality.^1^, ^2^

Although early stage melanoma is curable with surgical resection alone, it is an aggressive malignancy that tends to metastasize beyond its primary site; until the recent introduction of novel therapies the 5‐year survival rates of advanced melanoma were very poor (ranging from 5% to 19%).^3^

In fact, patients with metastatic melanoma (MM) are highly refractory to conventional chemotherapies and survival improvements have been not relevant with these therapies.^4^

In the previous years novel target therapies and immunotherapies improved overall survival (OS) and progression free survival (PFS) rates (ranging from 37% to 55%).^5^, ^6^ However only a part of MM patients demonstrate to have benefits and patient selection has become imperative. One of the reasons is that melanoma is one of the most complex cancers and the main concern remains about intra‐tumor heterogeneity (ITH).^7^

Fine needle aspiration (FNA) and core biopsy in target lesions are commonly used to confirm MM. In addition, histochemical and molecular analysis could provide potential biomarkers for patient stratification and monitoring therapies.

However, samples obtained from these procedures might be insufficient to provide accurate information of the whole lesion, in particular when wide intratumor heterogeneity is present.^7^ Moreover, the current model represented by one sampling from a single metastatic site could not intercept all subclones generated because of the rapid evolution of the tumor over time. Not least, FNA has sampling limitations and samples used for cytologic analysis might be insufficient for further molecular analyses.^8^

Imaging modalities (ie, Ultrasound [US], Computed Tomography [CT], Magnetic Resonance Imaging [MRI], Positron Emission Tomography [PET], as well as hybrid modalities) have maintained over the time a crucial role in clinical practice for monitoring therapy even if until few years ago radiological images evaluation was based mainly on qualitative assessment and dimensional measurements.^9^

This happened mainly because diagnostic imaging has the advantages of being accurate, minimally invasive, reproducible and presents higher patient compliance for monitoring tumor evolution.

As a consequence, several structured imaging criteria for different modalities have been proposed and continuously updated to distinguish responder from not responder patients.^10^, ^11^

Nowadays, imaging can potentially address further valuable information for personalized medicine that aims to predict the treatment outcome and tailor treatment strategy based on the characteristics of individual patients’ tumors.^12^, ^13^

Thanks to the technological advances registered in the previous years, different types of new image‐based quantitative measurements are now available, therefore both radiological and nuclear imaging might assume a more relevant role for monitoring novel therapies.

In addition, with the advent of targeted and immunotherapy treatments, a multidisciplinary/multimodality approach is becoming mandatory to personalize therapy and increase patient outcome.^14^

Regarding the image modalities generally adopted in the management of cutaneous melanoma patients, US examination has its major role in the follow‐up with limitations in the evaluation of therapy response principally because it is user‐dependent and cannot be used for lesion size measurement.^11^ US is useful to evaluate the surgical scar of the primary tumor, the in‐transit area, and the loco‐regional lymph nodes (LN) including LN basins.^15^

Whole‐body CT is a sensitive procedure that permits detection of metastases as small as 2‐4 mm and it continues to play a pivotal role during follow‐up of patients with advanced melanoma (stage IV) or in cases of suspected metastasis.^15^ Moreover, CT demonstrated to have a higher sensitivity compared to MRI in the diagnosis of small pulmonary metastases.^16^ The major drawbacks of CT are its limited soft tissue contrast and radiation exposure.

MRI in metastatic melanoma (MM) is the most widely used for determining the presence of brain metastases because of superior sensitivity to CT/PET‐CT for small lesions identification and their precise anatomical site evaluation.^15^, ^17^, ^18^ Whole‐Body MRI with diffusion‐weighted imaging in bone metastases could play an important role in the diagnosis of bone solid tumor metastases.^19^

PET has limitations that deserve consideration: among all it shows up areas of relevant uptake referred to inflammatory conditions that might be mistaken for cancers.

However, hybrid PET/CT examinations showed a superior sensitivity in detecting more visceral and non‐visceral metastasis than single modality.^13^, ^15^

The above modalities may have an increasingly relevant role in the assessment of treatment response, according to the localization of tumor and metastatic disease although most widely shared criteria (ie RECIST, PERCIST) are currently based principally on CT and PET imaging, respectively.^10^

### 1.1 Novel therapeutic options in the precision medicine era

Before the introduction of personalized medicine approach for cancer treatment management, treatments generally followed standardized protocols and response was often unpredictable with great variability between patients, in most cases unexplainable.

The introduction of novel target therapies and immunotherapies is changing the landscape of oncology opening new frontiers to conjugate imaging and predictive and prognostic biomarkers.^20^

Immunotherapy approaches with immune checkpoint inhibitors (ICIs) have shown important advances in the prognosis of metastatic melanoma,^21^ especially with the development of anti‐CTLA‐4 (ie Ipilimumab)^22^ and anti‐PD‐1 (ie Pembrolizumab and Nivolumab).^23^

In particular, Nivolumab has proven to significantly improve overall survival and progression free survival with a response rate of 30% in metastatic melanoma patients, meaning that patient selection is essential.^24^

However, the immunotherapy outcome is strongly influenced by tumor microenvironment, immune response and tumor molecular profiling.^25^

Moreover, immunotherapies could generate atypical patterns of response, different from those observed with drugs as chemotherapy and targeted therapies, that may not be properly evaluated by response criteria.^26^

In fact, an immune response can create the likeness of disease progression, as an increase of tumor size may not be representative of progression.^26^

Nevertheless, the development of immune‐specific related response criteria, RECIST 1.1 with its limitations remains the most shared method to assess therapy response.^27^

In this scenario is mandatory to develop novel more robust biomarkers predictive of treatment response capable to overcome imaging criteria limitations.^26^

However, medical images already contain further information hidden from visual assessment which can be measured. New quantitative approaches to image analysis are now possible, allowing the investigation of new biomarkers of imaging.

Radiomics is a new emerging field in radiology able to extract measurable features from biomedical images that might allow to respond to precision medicine needs.

Radiophenotypical data obtained by radiomics might be integrated by the genomic data (ie those obtained by new emerging liquid biopsy^28^) in a novel radiogenomic approach in order to further improve clinical decision process.

### 1.2 Radiomics

Radiomics is a novel high throughput quantitative imaging multi‐step process.

Main radiomics steps are: acquisition of medical images, extraction of a large number of quantitative imaging data called features and correlation of these with different endpoints.^29^, ^30^

In detail, after acquisition the lesions are delineated in images manually or using automated techniques (procedure called segmentation); successively quantitative parameters related to texture, shape and intensity of the lesions are extracted with different statistical orders.^29^

Texture analysis (TA) is one of the most widely spread radiomics methods where only the analysis of the textural properties of the images is taken into account.^31^

Radiomics features extraction is a not invasive step able to provide data over the volume of each lesion at multiple time points.^29^

As a result, radiomics and texture analysis(TA) can be complementary to the analysis of tissue samples that are achieved generally only once in one target lesion of a single anatomical site.

The last step of radiomics is to build classifier or mathematical models which are capable of providing prognostic information.

For example, a classifier can be used to stratify patients according to the radiophenotypical characteristics of a specific tumor potentially predictive of response in order to choose the best available therapeutic option.

The techniques for classifier development and validation are described in literature.^32^

To quantify the ability of the model to distinguish between classes (e.g, stratify patients) the parameter area under curve (AUC) is used.

AUC can assume a value from 0 to 1 (1 perfect separation between classes, 0 the classifier fails completely).

In this manuscript, we reviewed the state of the art of the application of radiomics/TA based on morphological and functional imaging in MM patients.

## 2 MATERIALS AND METHODS

### 2.1 Systematic search strategy and study selection criteria

The research questions for this systematic review are described as follows:

‘‘What are the known studies linking metastatic melanoma and radiomics/TA based on radiological/nuclear imaging?" "Which of these investigate either new radiomics‐based approaches or radiomic technological advancements hypothesizing their added value in novel treatment management of metastatic melanoma?"

A comprehensive literature search to identify relevant studies was done up to 10 September 2019 and it was restricted to the last 5 years using the following databases: MEDLINE/PubMed (National Center for Biotechnology Information, NCBI), EMBASE (Ovid) and the Cochrane Central Register of Controlled Trials (CENTRAL),Cochrane Library. The search string contains Medical Subject Headings (MeSH) and free‐text terms.

The included key search terms were; "neoplasms", "melanoma", "radiomics", "texture analyses", and "texture parameters". In order to include the maximum number of relevant papers we do not include key words regarding new therapies or precision medicine given the novelty of the topic.

The search method is completely described in the Supplementary Appendix.

The Preferred Reporting Items for Systematic Reviews and Meta‐Analyses (PRISMA) methodology was used for selecting studies based on the following criteria.

Only studies that met the following inclusion criteria were included; (a) only full ‐text papers in English language were considered, (b) the study population consisted only of humans and it was comprised of patients with cutaneous metastatic melanoma, radiomics analysis was performed only on radiological (CT, US, MRI) and nuclear medicine (ie PET, PET‐CT, SPECT) procedures.

Further selection was performed by applying the following exclusion criteria: (a) studies not using radiomics methodologies for treatment management, (b) and case reports, (systematic) reviews, and expert opinion papers and (c) trials that do not present at least partial results. Titles and abstracts were independently reviewed by two authors in order to decide study inclusion. In case of controversial judgment, the article was evaluated by a third author. Full articles were retrieved when the abstract was considered relevant. The bibliographies of retrieved papers were also evaluated to identify other relevant articles to be included.

**Figure 1:**
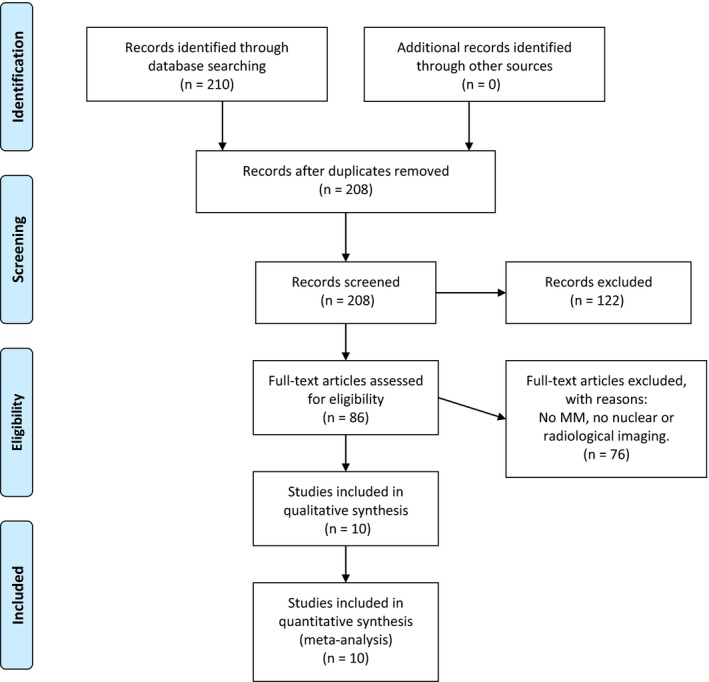
PRISMA flowchart reporting the search strategy adopted in this study

The PRISMA flowchart (see Figure 1) summarizes the adopted searching strategy used in this study.

### 2.2 Data extraction

A thorough systematic literature search and outcome extraction was independently performed by two authors (AG and EL).

From the included articles, the number of enrolled patients, the number of MM lesions, treatment type, the used imaging procedure (CT, PET, SPECT, MRI), the study endpoints (diagnosis/assess of therapy response), the main results of radiomics/TA, the adopted analysis (2D/3D), the radiomics software adopted for the analysis were extracted and tabulated; the reasons for study exclusion (if appropriated) were extracted and then registered.

In addition, most relevant statistical results (ie, *P*‐values, AUC) were extracted and described.

## 3 RESULTS

Overall, based on the previously specified search terms, 210 records have been identified until 10‐09‐2019; 78 studies were retrieved from MEDLINE/PubMed, 129 from Embase and three from CENTRAL database. Of these, 122 did not meet inclusion criteria and were excluded (mostly not full‐text article). Seventy‐six studies were excluded since they were not regarding MM, do not use radiomics analysis either on radiological or nuclear medicine imaging procedures (eg, dermatoscopy, confocal). Only one trial was identified in CENTRAL database but it was excluded because it did not report any results.

A total of 10 records were included.

The main results of the selected studies included in our analysis are reported in Table1.

**Table 1:** Relevant data and radiomics results reported in the selected studies

Author, reference N. and year	Image modality	N. MM patients	*Tot *N. of patients	treatment	Study end‐point	Results of Radiomics & texture analysis	Type of approach	Radiomics software
Saadani^33^ (2019)	PET	35(100 lesions)	35	NA	BRAFV600 mutation correlation with PET radiomics features	BRAFV600 was not predicted by radiomics or conventional PET features	2D & 3D	In‐house
Sun^34^ (2019)	CT	45	NA	Anti‐PD‐1	Radiomics‐based biomarker implementation for immunotherapy	The developed radiomic signature was a predictor of immunotherapy response	2D & 3D	LifeX
Trebeschi^35^ (2019)	CT	80 (483 lesions)	203	Anti‐PD‐1	Immunotherapy response/OS	Greater morphological heterogeneity was significantly associated with immunotherapy response	2D & 3D	Python package
Della Seta^36^ (2019)	MR	21	48	SRT	OS, PFS	High level enhancement tumor volume was associated with longer OS and IPFS	3D	IntelliSpace Portal V.8, Philips Healthcare
Kniep^37^ (2019)	MR	26 (69 lesions)	189	baseline	Feasibility study	Three‐class model produced the highest area under curve of model including age, sex and image features	3D	Python package
Durot^38^ (2019)	CT	31	31	Pembrolizumab	OS, PFS	Skewness (>−0.55) was significantly associated both with lower OS and PFS	2D	TexRAD
Ortiz‐Ramon^39^ (2018)	MR	23 melanoma lesions	38	baseline	Feasibility study	3D MRI texture features were usable for the differentiation of brain metastasis	2D & 3D	Radiomics (Matlab)
Ortiz‐Ramon^40^ (2017)	MR	23 melanoma lesions	30	NA	Classification model	Five 3D predicting models better than 2D	2D & 3D	Radiomics (Matlab)
Giesel^41^ (2017)	FDG, Ga‐DOTATOC and Ga‐PSMA PET/CT vsCTradiomics	33 (224 lymph nodes)	148	NA	Classification model and lymph nodes staging	Correlation between PET and CT extracted data in mm patients	NA	Software developed at the Fraunhofer Institute for Medical Image Computing
Smith^42^ (2015)	CT	40	42	Bevacizumab	OS	CT images, a model incorporating CT texture analysis of target lesions, tumor size changes, and baseline LDH levels were highly accurate in predicting OS	2D	TexRAD (version 1.0.5, TexRAD)

In particular, almost all the papers from our research date back from 2017 to 2019 (N = 9) and one in 2015; a large number of these (N = 6) have been published in 2019.

The investigated image modalities were MRI (N = 4 studies), CT (N = 4 studies), in one study PET/CT and another only PET imaging. The patient number ranged from 21 to 80 patients per study, while the number of lesions varied from 23 to 483. None of the studies selected from our searching investigated US imaging.

The study endpoints were overall survival (OS) (N = 3 studies), progression free survival (PFS) (N = 2 study), response to therapy (N = 1) or development of a classification model (N = 4 studies). The software used for radiomics/TA were PyRadiomics Python package (N = 2 study), TexRAD software (N = 2 studies), the IntelliSpace Portal V.8 Philips Healthcare, LifeX software (N = 1 study) the MATLAB toolbox Radiomics implemented by Vallieres et al (N = 2 studies).^43^ In one study, the radiomics/TA was made with a software developed at the Fraunhofer Institute for Medical Image Computing.^44^ In another study an in‐house built software was used for radiomics analysis.^33^

The features were extracted using a 2D (N = 2 studies) or 3D (N = 3 studies) or 2D & 3D (N = 5 studies) approach, while no details were provided in one study.

Out of 10 studies, five investigated radiomics methods as predictors of response to therapy.

Trebeschi et al^35^ tried to demonstrate that radiomics‐based biomarkers can automatically quantify radiographic characteristics. They concluded that they could be used as predictors of immunotherapy response.

Their population was composed of both NSCLC and melanoma patients treated with immunotherapy.

The radiomics‐based classifier was developed using machine learning techniques.

Greater morphological heterogeneity was found in association with response among most common melanoma lesions.

A subanalysis on different anatomical sites was made. Increased values of morphological heterogeneity in hepatic, nodal and splenic lesions was associated with response.

Della Seta et al^36^ evaluated the feasibility of use quantitative tissue enhancement (QTE) of brain metastasis in pre‐treatment brain MRI as a radiomic biomarker for therapy response in patients treated with SRT.

QTE values for the patients showed significant results in a univariate and multivariate analysis for OS (HR = 0.375 for univariate and HR 0.376 for multivariate respectively).

In univariate analysis they showed longer PFS rates (HR = 0.046).

They reported a cut‐off threshold of 68,61% for QTE. They concluded that above this QTE value patients survived significantly longer (4.9 vs 10.2 months).

Durot et al^38^ investigated whether texture analysis of pre‐treatment contrast‐enhanced CT images could predict immunotherapy response (ie, Pembrolizumab).

They used OS and PFS as endpoints.

They report that the feature skewness, which is calculated from the histogram of the images, is correlated with OS and PFS. Skewness is a quantitative measure that can be extracted by texture analysis dedicated software, representative of the heterogeneity of a segmented lesion.

They found that above a threshold value of −0.55 skewness was associated with lower OS and PFS in patients treated with Pembrolizumab.

They reported skewness as an independent predictor of OS and PFS and concluded that TA might be useful for pre‐therapy patient selection.

The purpose of the study of Smith et al^42^ was to explore the use of CT TA to predict treatment response on initial post‐therapy images in MM patients treated with anti‐angiogenetic drug (Bevacizumab).

Pre‐ and initial post‐therapy images were analyzed to demonstrate if the variation of the radiomic features might be a predictor of response (Delta radiomics).

The absolute change of the feature mean positive pixel (MPP, the average of all the positive pixel in the ROI) was correlated with OS (Hazard‐Ratio [HR] of 5.05 for decrease in MPP versus increase).

They found that patients treated with Bevacizumab showed a fivefold greater risk of mortality when a decrease in a radiomic feature (MPP) was present on initial posttherapy CT.

They concluded that a prognostic index comprehensive of MPP absolute change, LDH baseline level and tumor size was a predictor of OS.

Sun et al^34^ developed a radiomic‐based signature of tumor infiltrating CD8 cells inpatient treated with anti‐PD1 and validated it in three independent cohorts.

They found that in the cohort which includes melanoma patients the radiomic signature at baseline was a predictor of treatment response. They reported that the higher values of radiomic signature at baseline significantly correlated with longer survival.

Other three studies of the total records, investigated the development of a model for secondary lesion characterization and differentiation.

In detail, Kniep et al^37^ evaluated the feasibility to predict tumor type by analyzing brain metastasis using routine MRI and machine learning techniques. The analysis focused on brain metastasis from melanoma, breast and NSCLC.

The authors developed and validated a radiomic‐based biomarker. The developed classifier performed well when it included even clinical data (age and sex) with a reported AUC = 0.83 for MM.

The classifier results were also compared with radiologists’ reading performance; better performances of the classifier were reported for MM.

Ortiz‐Ramon et al^40^ evaluated the capability of an MRI radiomics‐based classifier to identify the primary tumor site of origin (ie, melanoma and lung cancers) of brain metastasis. The authors used five different predictive models to evaluate the discriminative power of radiomic features. They reported an AUC higher than 0.846 for every method for 2D features and higher than 0.925 for 3D features.

The authors also showed that the 3D approach in radiomics analysis performed better than 2D.

In the other paper by Ortiz‐Ramon^39^ the classifier was built using MRI images of brain metastasis of patients with melanoma, breast and lung cancer. The model built using eight features was able to differentiate lung brain metastasis from melanoma metastasis with a reported AUC of 0.936. The classification performances of breast brain metastasis versus melanoma brain metastasis were poorer (AUC = 0.607).

Giesel et al,^41^ tried to demonstrate that radiomics CT features are complementary to the analysis of SUVmax in Ga68‐DOTATOC, 68Ga‐PSMA and FDG‐PET sentinel lymph node procedure.

They found that CT densities are correlated with the PET uptake. A threshold of 7.5 HU was reported to be discriminatory between malignant and benign LNs infiltration. A value of 20 Hounsfield units was indicative to exclude benign LN.

Saadani et al^33^ evaluated the feasibility to assess the mutation status of the metabolic biomarker BRAFV600 by the use FDG‐PET radiomic and conventional features (eg, SUVmax).

A correlation of BRAFV600 mutation with PET features was not found; they reported a low prediction power of radiomic features (AUC = 0.62) and they found no correlation between conventional features and BRAFV600 mutation.

## 4 DISCUSSION

Imaging‐based quantitative data have been assuming a more relevant role in staging, restaging, and therapy assessment resulting in potential minimally invasive prognostic biomarkers useful for a personalized medicine approach.

In the precision medicine era, novel therapies generate radiologic patterns different from conventional ones. For example, treatment response after immunotherapy can be associated with pseudo‐progression or flair phenomena,^45^ meaning that the enlargement of old lesions and the appearance of new lesions soon after treatment may not reflect real disease progression.^26^

In addition, activation of the immune system to fight cancer may lead to unwanted autoimmune‐mediated toxic effects that may generate atypical patterns could be mistaken for progression disease or misdiagnosed delaying appropriate clinical management.^46^

Thus, individual medicine has become mandatory to predict treatment response early.

In this scenario, radiomics offers the opportunity to look beyond the images extrapolating new data that may be use as imaging biomarkers.^29^, ^30^ Among different fields of application, oncology is the one where radiomics presents more promising opportunities.

Our systematic review focuses on the predictive and prognostic value of image‐based radiomics/TA approach in MM patients in order to assess the added value of this novel methodology.

For example, by applying the Radiomics/TA approach on CT images, it is possible to quantify a series of quantitative parameters such as baseline and initial posttherapy changes in MM patients 38, 42 with target lesion. TA features have been already associated with survival in patients with head and neck cancer,^47^ colorectal cancer,^48^ esophageal cancer,^49^ non‐small cell lung cancer.^50^

In addition, as reported for other tumors,^47^, ^48^, ^49^, ^50^ radiomics/TA findings could also reflect in MM patients^38^, ^42^ a wider range of tumor biologic data including tumor angiogenesis, hypoxia, blood flow, glucose metabolism, necrosis and tumor heterogeneity at microscopic levels.

Moreover, radiomics represents a cheap and easy‐to‐implement strategy to be used in clinical practice from baseline to tumor recurrence.

Melanoma is one of the most heterogeneous tumors, which is highly aggressive at advanced stage.^7^ Radiomics gives a representation of tumor phenotype that could be integrated with other data (eg, molecular) in order to provide useful information in supporting clinical decision process.^29^

We found a limited number of studies about radiomics/TA using our search terms likely due to the difficulty in recruiting a large cohort of metastatic melanoma patients or retrieving a homogeneous cohort of these patients for a retrospective study.

Moreover, the very recent diffusion of radiomics methodology contributed to the limited number of studies published on this issue. Despite this, it is worthwhile to notice that most of the papers were published in the last two years, indicating an increasing interest in radiomics.

The results of the included studies are heterogeneous in terms of aim, diagnostic imaging procedures used and radiomics analysis.

Anyway, half of them (N = 5) investigated the use of radiomics to find imaging biomarker for prediction of treatment response.

Of these, Trebeschi et al found that lesions with a more heterogeneous morphological profile are more likely to respond to immunotherapy giving a biological rationale. Despite their findings suggesting an association between the radiomics features expression of tumor radiophenotypic pattern and immunotherapy response, the melanoma cohort was too small to identify robust imaging biomarkers.^35^

More data analysis/collection and studies on large cohorts are needed for this purpose.

A machine learning method should be used to improve the process of patient stratification by implementing new diagnostic models. However, the machine learning model proposed by Trebeschi et al performed poorly for the melanoma dataset.

Della Seta et al proposed a radiomic 3D quantitative measurement of tissue enhancement in the baseline MRI to predict stereotactic radiation therapy response showing that a higher percentage of enhancement is a predictor of longer survival.^36^ The results are statistically significant, however there is no mention about reproducibility of this radiomic feature. In fact, reproducibility is one of the most relevant challenges for routine use of radiomics.^30^

In a recent study of Durot et al^38^ pre‐treatment CTTA‐derived tumor skewness has been reported as possible predictive biomarker of OS and PFS in MM patients who underwent anti‐PD1 monoclonal antibody therapy.

Authors took into account only a subgroup of reproducible features. However, the subset of features analyzed has been already demonstrated to be predictive of response in other type of cancer.^51^ The strength of this study is the creation of a simple model using a limited number of more robust features.

Smith et al^42^ found that the variation of the feature means a positive pixel is significantly correlated with response in patients treated with antiangiogenetic drugs.

In particular, they focused their analysis on patient with RECIST stable disease given the high variability of survival that these subgroup of patients have.

The added value of this work is the delta radiomics approach. This kind of analysis highlights the radiophenotypic patterns at different time points reflecting tumor heterogeneity evolution during treatment.

A prognostic index comprehensive of radiomic feature, baseline LDH and tumor size was developed to predict OS in targeted therapy.

Sun et al^34^ developed and validated a radiomic signature which is significantly correlated with treatment response in patients treated with immunotherapy.

Their work is very interesting for various reasons: the large number of cohorts and patients involved, the methodologies used and the significative final results. Indeed, they developed and validated the radiomic signature using three independent cohorts.

However, the cohorts were not uniform in terms of image acquisition parameters and this constitutes a weakness for the robustness of the radiomic signature.

Three studies concentrated on the role of radiomic‐MRI based approach in identifying the primary tumor site by analyzing metastasis and in particular all these studies took into account brain metastasis.

Although a relatively small percentage of patients have brain metastasis, melanoma is one of the tumors that more frequently metastasizes to the brain (5%‐20%)^39^, ^40^ and MRI is highly recommended to detect and characterize brain lesions. Quantitative TA may add useful information to those acquired with MRI. Different TA approaches 2D/3D have been investigated showing the superiority of 3D approach for predicting treatment response^39^, ^40^ as well as to develop a classification model.^37^

Kniep et al. developed a model which was able to able to reveal the primary tumor site by analyzing brain metastasis MRI images using a radiomic approach.

The prediction performance of the built model was also compared with the reading performance of two experienced radiologists.

The classifier performed better than the radiologists for all the primary tumor site and for melanoma it showed the highest discriminative power, likely due to the peculiar characteristic of melanoma lesions to be high in melanin, which may influence the MRI signal.

Ortiz‐Ramon et al. in their papers developed and validate several models using MRI features extracted from brain metastasis to predict the primary tumor. In one study by Ortiz‐Ramon et al^40^ the classifier performed well in distinguishing between melanoma and lung cancer brain metastasis. In their other study,^39^ they reported the worst performance to distinguish between melanoma metastasis and brain metastasis from either breast or lung cancer.

Nuclear imaging‐based radiomics has more limitations with respect to other radiological modalities (eg, CT, MRI). This might be due to the lower resolution and contrast of nuclear medicine images.^33^

In the study by Giesel et al,^41^ only LNs were evaluated in order to discriminate those with metastatic infiltration from benign ones. Despite the good results reported, the robustness of radiomic features extracted from small volumes of LNs was overlooked. In fact, radiomic may fail to provide significant results when the region of interest of a segmented lesion on the image is composed of a few number of pixels.

Saadani et al^33^ tried to correlate BRAFV600 mutation with conventional and radiomic PET features. However, they did not take into account the limitations of low spatial resolution and low contrast. As a result, the study was not satisfactory in terms of discriminative power.

Anyway, the relevant role of both PET and PET/CT examinations is well demonstrated for the surveillance of distant metastases in melanoma patients with high sensitivity (86%) and specificity (91%).^52^ However, the prognostic and predictive role of quantitative 18F‐FDG‐PET analysis is debated and it remains controversial for monitoring therapies.^53^, ^54^ Radiomics in nuclear imaging might add useful information to make the quantitative PET data more robust. However, the use of radiomics in nuclear imaging is an open challenge.

Radiomics could provide new quantitative imaging biomarkers to support MM management. These novel imaging biomarkers might be correlated with a panel of diagnostic, prognostic and predictive biomarkers, either immunohistochemical (ie PD‐L1), serological (ie Lactate Dehydrogenase, LDH) and molecular (ie tumor mutational burden, TMB), proposed in literature^55^, ^56^ for support the management of advanced stage melanoma patients.

To date, many of these findings require further studies and independent validation.

Despite the promising clinical potential of radiomics, there are precautions that must be taken in order to consider the prognostic power of radiomic features.

An appropriate methodological approach needs to be invoked in order to select robust and reproducible data.^29^

Multicentric studies with larger cohorts are needed to  validate radiomic‐based imaging biomarkers.

Ad hoc prospective studies need to be planned and conducted in MM patients to reveal the additional value of radiomics to the qualitative assessment of morphological and functional imaging.

## 5 CONCLUSION

Here, we have reviewed the applications and challenges of radiomics in MM patients. Radiomics and TA may represent a novel robust strategy demanding further investigation in order to quantify various tumor phenotypes on medical images, may be related to genetics and clinical outcomes. The preliminary results are encouraging. However, larger cohorts and more homogeneous data are recommended to provide definitive and robust results. Machine learning models to support clinical decision needed to be implemented on more reproducible data.
