# Supplementary material for: Novel cancer therapies for advanced cutaneous melanoma: The added value of radiomics in the decision making process–A systematic review
Source: Cancer Med. 2020 Jan 17;9(5):1603–12. doi: 10.1002/cam4.2709 (PMC7050080; doi:10.1002/cam4.2709)
Supplement: Supplementary file 1 [file CAM4-9-1603-s001.docx]

**Appendix 1**

**Search methods**

The research has been done on three databases: MEDLINE/PubMed (National Center for Biotechnology Information, NCBI), EMBASE (Ovid) and the Cochrane Central Register of Controlled Trials (CENTRAL) until 10-09-2019.The search string contains free-text and/or Medical Subject Headings (MeSH) search of 5 key search terms: "neoplasms", "‘melanoma", "radiomics", "texture analyses and "texture parameters" . In order to include the maximum number of relevant papers we do not include key words regarding new therapies or precision medicine given the novelty of the topic.

PUBMED

**#1** "Neoplasms"[Mesh:NoExp]

**#2** "Melanoma"[Mesh] OR Melanoma[Title/Abstract]

**#3** #1 OR #2

**#4** Radiomic*[Title/Abstract] OR “texture analysis” [Title/Abstract] OR “texture parameters” [Title/Abstract] OR “texture parameter” [Title/Abstract]

**#5** #3 AND #4

**#6** #5 NOT animals[MeSH Terms] NOT (animals[MeSH Terms] AND Humans[MeSH Terms])

**#7** #6 NOT (Letter [pytp] OR Case Reports [pytp] OR Editorial [pytp])

EMBASE

**#1** 'neoplasm'/de

**#2** 'melanoma'/exp OR melanoma

**#3** #1 OR #2

**#4** radiomic*:ti,ab OR 'texture analysis':ti,ab OR 'texture parameters':ti,ab OR 'texture parameter':ti,ab

**#5** #3 AND #4

**#6** #5 NOT ([animals]/lim NOT [humans]/lim)

**#7** #6 NOT ('editorial'/it OR 'letter'/it OR 'case reports'/it OR 'review'/it)

CENTRAL

#1 MeSH descriptor: [Neoplasms] this term only

#2 MeSH descriptor: [Melanoma] explode all trees

#3 melanoma

#4 #2 OR #3

#5 (radiomic* OR "texture analysis" OR "texture parameters" OR "texture parameter")

#6 #1 OR #4

#7 #6 AND #5 with Publication Year from 2015 to 2019, in Trials
